# Inhibition of DPP-4 reduces acute mortality after myocardial infarction with restoration of autophagic response in type 2 diabetic rats

**DOI:** 10.1186/s12933-015-0264-6

**Published:** 2015-08-11

**Authors:** Hiromichi Murase, Atsushi Kuno, Takayuki Miki, Masaya Tanno, Toshiyuki Yano, Hidemichi Kouzu, Satoko Ishikawa, Toshiyuki Tobisawa, Makoto Ogasawara, Keitaro Nishizawa, Tetsuji Miura

**Affiliations:** Department of Cardiovascular, Renal and Metabolic Medicine, Sapporo Medical University School of Medicine, South-1, West-16, Chuo-ku, Sapporo, 060-8543 Japan; Department of Pharmacology, Sapporo Medical University School of Medicine, Sapporo, 060-8543 Japan

**Keywords:** Type 2 diabetes, Autophagy, DPP-4 inhibitor, Myocardial infarction, Mortality

## Abstract

**Background:**

Type 2 diabetes mellitus (T2DM) worsens the outcome after myocardial infarction (MI). Here, we hypothesized that inhibition of dipeptidyl peptidase-4 (DPP-4) improves survival after MI in T2DM by modifying autophagy in the non-infarcted region of the heart.

**Methods and results:**

Under baseline conditions, there was no significant difference between levels of myocardial autophagy marker proteins in OLETF, a rat model of T2DM, and in LETO, a non-diabetic control. However, in contrast to the response in LETO, LC3-II protein and LC3-positive autophagosomes in the non-infarcted region of the myocardium were not increased after MI in OLETF. The altered autophagic response in OLETF was associated with lack of AMPK/ULK-1 activation, attenuated response of Akt/mTOR/S6 signaling and increased Beclin-1–Bcl-2 interaction after MI. Treatment with vildagliptin (10 mg/kg/day s.c.), a DPP-4 inhibitor, suppressed Beclin-1–Bcl-2 interaction and increased both LC3-II protein level and autophagosomes in the non-infarcted region in OLETF, though it did not normalize AMPK/ULK-1 or mTOR/S6 signaling. Plasma insulin level, but not glucose level, was significantly reduced by vildagliptin at the dose used in this study. Survival rate at 48 h after MI was significantly lower in OLETF than in LETO (32 vs. 82%), despite similar infarct sizes. Vildagliptin improved the survival rate in OLETF to 80%, the benefit of which was abrogated by chloroquine, an autophagy inhibitor.

**Conclusions:**

The results indicate that vildagliptin reduces T2DM-induced increase in post-MI acute mortality possibly by restoring the autophagic response through attenuation of Bcl-2-Beclin-1 interaction.

**Electronic supplementary material:**

The online version of this article (doi:10.1186/s12933-015-0264-6) contains supplementary material, which is available to authorized users.

## Background

Diabetes mellitus (DM) is associated with poor outcome after acute myocardial infarction (MI) even in the era of reperfusion therapy [1, 2]. The poor outcome after MI in patients with DM has been explained by extensive atherosclerotic lesions, increased myocardial susceptibility to ischemia/reperfusion injury, and contractile dysfunction of the myocardium, often called “diabetic cardiomyopathy” [3]. Augmentation of contractile function in the non-infarcted region is crucial in acute compensation for the lost contraction in the infarct region. Thus, pre-existing contractile dysfunction in patients with DM, if any, potentially compromises such a compensatory response after MI and increases mortality. Recently, we confirmed DM-induced increase in acute mortality after MI in an animal model of obese type 2 DM (T2DM), Otsuka-Long-Evans-Tokushima Fatty rats (OLETF). The mortality rate at 48 h after MI was significantly higher in OLETF due to lethal heart failure than in non-diabetic control rats, Long-Evans-Tokushima-Otsuka rats (LETO), while infarct sizes were similar in OLETF and LETO [4]. Interestingly, OLETF had preserved ventricular contractility with mildly impaired relaxation under baseline conditions [4]. The findings indicate that the altered response of the non-infarcted myocardium, rather than baseline ventricular contractility, contributes to the T2DM-induced increase in acute mortality after MI.

Autophagy is a cellular process of lysosome-mediated degradation of cytoplasmic components or damaged organelles in response to cellular stress [5–13]. It has been suggested that autophagy has roles in critical adaptive mechanisms in the heart under hemodynamic stress conditions such as pressure overload [6] and loss of a contractile region by MI [5, 7–11]. In the case of MI, autophagic activity was augmented in the non-infarcted remote area and border area but not in the infarcted myocardium after MI, and the autophagic activity progressively increased in the remote area during a 3-week period after MI [8, 9]. The increase in autophagic activity has protective effects against remodeling and dysfunction of the ventricle after MI [8, 9]. However, autophagy has been shown to be impaired by DM in non-cardiac [14] and cardiac tissues [15–20], though advanced glycogen endproducts reportedly activate autophagy in cardiomyocytes [21]. Whether the increase in myocardial autophagic activity after MI is impaired by DM and whether such an impairment, if any, is treatable by pharmacological agents have not been clarified.

We hypothesized that autophagic response of the myocardium to MI is impaired by T2DM and that inhibition of dipeptidyl peptidase-4 (DPP-4) would attenuate the T2DM-induced increase in post-MI mortality by restoring the autophagic response. The rationale for the hypotheses is four-fold. First, a significant association of preserved ventricular function and activation of autophagy has been demonstrated for different types of cardiac stress in non-diabetic animals [5–11]. Second, DM impairs intracellular signaling mechanisms relevant to autophagy, including PI3K-Akt signaling, in the myocardium [3]. Third, activation of the glucagon-like peptide-1 (GLP-1) receptor or treatment with a DDP-4 inhibitor triggers AMP-activated protein kinase (AMPK) signaling [22, 23, 24], which facilitates autophagy [5, 9, 15, 18, 25]. In fact, a GLP-1 analog, liraglutide, has been shown to promote autophagy in non-cardiac cells [26]. Fourth, it has been reported that the activity of circulating DPP-4 is associated with left ventricular dysfunction in patients [27, 28]. Conversely, inhibition of DPP-4 and use of a GLP-1 analog prevented cardiomyopathy, improved cardiac function and post-MI survival rate, and attenuated ventricular remodeling in DM and non-DM animals [27, 29–32]. To examine the hypotheses, we used OLETF as a model of T2DM in the present study as in previous studies [4, 33, 34]. Results of the experiments showed that a DPP-4 inhibitor, vildagliptin, significantly improved survival after MI in OLETF and that the protective effect of vildagliptin was closely associated with restoration of the autophagic response in the non-infarcted myocardium.

## Methods

### Animals and experimental protocol

**Fig. 1:**
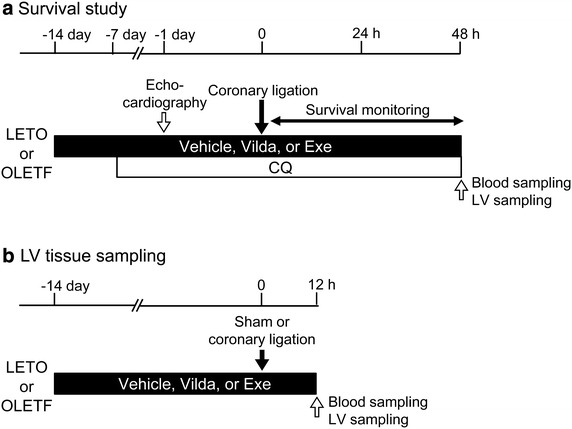
Experimental protocols. Experimental protocols for survival study (**a**) and cardiac tissue sampling (**b**). *Vilda* vildagliptin, *Exe* exenatide, *CQ* chloroquine, *LV* left ventricle.

The present study was conducted in strict accordance with the Guideline for the Care and Use of Laboratory Animals published by the US National Institute of Health (NIH publication No. 85-23, revised 1996) and was approved by the Animal Use Committee of Sapporo Medical University. Protocols of the experiments are summarized in Fig. 1. Male LETO and OLETF at ages of 25–30 weeks were used in all experiments. LETO were pretreated with saline, vildagliptin (10 mg/kg/day), a DPP-4 inhibitor, or exenatide (10 μg/kg/day), a GLP-1 receptor agonist, for 2 weeks, or with chloroquine (10 mg/kg/day), an autophagy inhibitor [9, 12, 35], for 1 week. Vildagliptin was kindly provided by Novartis Pharma AG (Basel, Switzerland). The pharmacological agents were administered via osmotic minipumps (Alzet, Cupertino, CA, USA), not via drinking water containing the agents, because the amount of water rats drink per day is not consistent. The dose of vildagliptin was selected on the basis of a result in a previous report [36], and we confirmed that this dose of vildagliptin significantly increased the serum level of GLP-1 (see “Results”). OLETF also received saline, vildagliptin, exenatide or chloroquine as did LETO, and an additional group of OLETF received both vildagliptin and chloroquine. In separate groups of rats, blood samples were collected via the carotid artery under anesthesia 12 h after fasting to examine the effects of these pharmacological agents on metabolic parameters.

### Oral glucose tolerance test

An oral glucose tolerance test (OGTT) was performed in LETO treated with a vehicle and OLETF treated with a vehicle or vildagliptin (10 mg/kg/day) for 2 weeks. After fasting for 12 h, rats were administered glucose (2 g/kg body weight) by gavage, and blood glucose and insulin levels before and after glucose administration were measured by using a Glutest-mint (Sanwa Kagaku Kenkyusho, Nagoya, Japan) and a rat insulin RIA kit (Linco Research Inc, St. Charles, MO, USA), respectively. Blood for GLP-1 assay using a GLP-1 (Active) ELISA kit (Millipore) was collected before glucose administration in sampling tubes containing a DPP-4 inhibitor.

### Echocardiography

Echocardiography was performed before induction of MI as previously reported [4].

### Induction of MI and mortality monitoring

Rats were prepared for induction of MI as in our previous study [4]. In brief, rats were anesthetized with sodium pentobarbital (40 mg/kg, i.p.), and the level of anesthesia was continuously monitored during the experiment and an additional dose of pentobarbital was administered when necessary. Rats were then intubated and ventilated with a rodent respirator (model 683, Harvard Apparatus, South Natick, MA, USA). After left thoracotomy, a marginal branch of the left coronary artery was permanently ligated by using a 5–0 silk thread to induce MI. We used a permanent occlusion model of MI to avoid the possibility that pharmacological pretreatments modify infarct size and induce an inter-group difference in mechanical stress on the non-infarcted region. The surgical wounds were repaired and the rats were returned to their cages. All rats were allowed ad-lib access to water but restricted from food for 12 h. Survival rate of rats was determined at 24 and 48 h after MI. Rats that had survived at 48 h after MI were euthanized by a pentobarbital overdose and heart tissue was excised and fixed in 10% formaldehyde for infarct size analysis.

### Cardiac tissue sampling after MI

Since the mortality rate at 24–48 h after MI was high in OLETF at ages of 25–30 weeks [4], myocardial tissue sampling for biochemical analyses and immunohistochemistry was performed at 12 h after MI. Rats were anesthetized and ventilated, and blood pressure and heart rate were monitored by a catheter placed in the carotid artery. The chest was re-opened and the hearts were excised and immediately immersed in ice-cold saline. The myocardium in the non-infarcted region was quickly excised in the saline, frozen in liquid nitrogen, and stored at −80°C until use for biochemical and histological analyses.

### Immunohistochemistry

Frozen heart tissues were embedded in OCT compound (Tissue-Tek) and snap-frozen in liquid nitrogen. After the tissues had been sectioned at 8 μm in thickness with a cryostat at −20°C, the sections were incubated with rabbit polyclonal anti-LC3 antibody (MBL, PM036, 1:250) in PBS containing 1% BSA and 0.3% triton X-100 overnight at 4°C. The samples were then incubated with an Alexa Fluor 488 anti-rabbit IgG antibody (Invitrogen) for 1 h at room temperature. After nuclei had been stained with Hoechst33342 (Dojindo, Kumamoto, Japan), samples were mounted on slides for image analysis. Fluorescence images were obtained using a FLoid Cell Imaging Station (Life Technologies). The number of LC3 dots was counted and analyzed in 40 randomly selected fields from five hearts in each group.

### Immunoblotting

Frozen tissue samples were homogenized in ice-cold buffer (CelLytic™ MT Cell Lysis Reagent) including protease and phosphatase inhibitor cocktails (Nacalai Tesque, Inc., Kyoto, Japan). The homogenate was centrifuged at 15,000*g* for 15 min at 4°C to obtain the supernatant. Equal amounts of protein were analyzed by immunoblot assays using specific antibodies (see Additional file 1: Table S1). Intensities of individual bands were quantified by using Image J software (National Institutes of Health).

### Beclin-1–Bcl-2 interaction

Frozen myocardial tissue samples were homogenized in ice-cold buffer containing 20 mM Tris (pH 7.4), 137 mM NaCl, 10% glycerol, 0.3% CHAPS, and 2 mM EDTA supplemented with protease and phosphatase inhibitor cocktails. The homogenates were then centrifuged at 15,000*g* for 15 min to obtain supernatants. After quantification of protein concentration, the lysates were incubated for 30 min with 40 μl of protein A magnetic beads (New England Biolads, Ipswich, MA, USA) to remove endogenous IgG. Equal amounts (2,000 μg) of lysates were incubated with either 6 μg of rabbit anti-Beclin-1 antibody or normal rabbit IgG overnight at 4°C. The mixture was then incubated with 50 μl of fresh beads for 1 h. The beads were washed three times with PBS containing protease inhibitor cocktail and re-suspended in SDS sample loading buffer followed by denaturation. Immunoprecipitated proteins were analyzed by Western blotting initially with rabbit anti-Bcl-2 antibody and then with mouse monoclonal anti-Beclin-1 antibody after stripping.

### mRNA quantification

Total RNA was isolated from myocardial tissues by using an RNeasy Fibrous Tissue Mini Kit (Qiagen, Valencia, CA, USA). First-strand cDNA was synthesized using a SuperScript VILO™ cDNA Synthesis Kit (Life Technologies). DNA amplification was performed in StepOne™ (Life Technologies). Analyses of B-type natriuretic peptide (BNP) and β-actin mRNA levels were performed by using Taqman gene expression assays (Rn00676450_g1 Nppb and Rn00667869_m1 Actb, respectively). For p62 and 18S, we used Power SYBR PCR Master Mix (Applied Biosystems, Inc) and the following oligonucleotide primers: for rat p62, 5′-ATCAGCCTCTGGTGGGAGAT-3′ and 5′-CCCATCCACAGGTGAACTCC-3′; for rat 18S, 5′-CGGACAGGATTGACAGATTG-3′ and 5′-CAAATCGCTCCACCAACTAA-3′. All assays were performed in duplicate and by the standard curve method using serial cDNA dilution.

### Statistical analyses

Data are presented as mean ± SEM. Differences between treatment groups were assessed by one-way analysis of variance (ANOVA) followed by the Student–Newman–Keuls post hoc test for multiple comparisons. Differences in time course between two groups in OGTT were analyzed by 2-way ANOVA for repeated measures followed by the Student–Newman–Keuls post hoc test for multiple comparisons. Survival rates after MI were compared by Kaplan–Meier curves and log-rank statistics. For all tests, p < 0.05 was considered statistically significant.

## Results

### Metabolic profiles

**Fig. 2:**
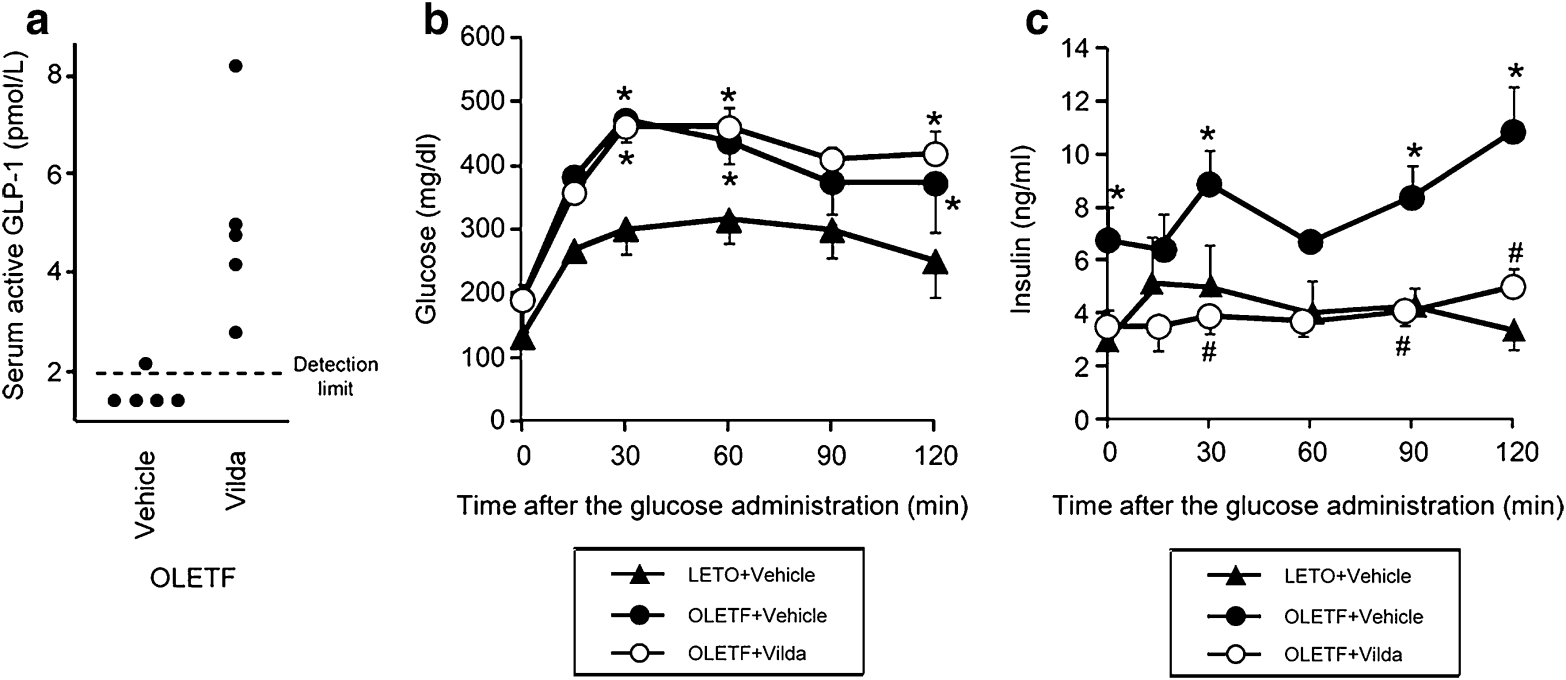
Serum active GLP-1 level and oral glucose tolerance test in LETO and OLETF. **a** Serum level of the active form glucagon-like peptide (GLP-1) in OLETF treated with the vehicle or vildagliptin. Values in 4 of 5 samples from the vehicle-treated group were under the detection limit (<2.0 pmol/L) of the assay. Blood glucose (**b**) and serum insulin (**c**) during an oral glucose tolerance test (2 g glucose per kg body weight) in LETO (*triangle*) and OLETF treated with the vehicle (*closed circle*) or vildagliptin (*open circle*). *p < 0.05 vs. LETO and ^#^p < 0.05 vs OLETF vehicle at each time point. N = 5 in each group. *Vilda* vildagliptin.

Data for metabolic profiles of LETO and OLETF and the effects of vildagliptin, exenatide and chloroquine on metabolic parameters are shown in Table 1. In LETO, body weight (531 ± 7 g), fasting blood glucose level and serum total cholesterol level were not changed by treatment with vildagliptin or exenatide. OLETF had larger body weight (627 ± 14 g) and higher fasting blood glucose level than those of LETO (Table 1), as we previously reported [4, 34, 37]. Treatment with vildagliptin or exenatide at the dose used in the present study did not change body weight and blood glucose level in OLETF. Serum insulin level was significantly higher in OLETF than in LETO, and both vildagliptin and exenatide decreased insulin level in OLETF without reduction in blood glucose level (Table 1). Treatment of OLETF with chloroquine in addition to vildagliptin affected neither insulin level nor blood glucose level compared to the levels in those treated with vildagliptin alone. Despite lack of effects on blood glucose level, vildagliptin increased serum level of the active form of GLP-1; GLP-1 level was below the detection range (<2.0 pmol/L) in 4 of a total of five samples from the vehicle-treated OLETF, but it was 5.0 ± 0.9 pmol/L in vildagliptin-treated OLETF (Fig. 2a).

**Table 1:** Metabolic parameters after treatment

	LETO			OLETF			
	Vehicle	Vildagliptin	Exenatide	Vehicle	Vildagliptin	Exenatide	Vildagliptin + Chloroquine
Blood glucose (mg/dl)	108 ± 5	106 ± 6	117 ± 12	205 ± 25*	201 ± 13*	251 ± 17*	197 ± 22*
Serum insulin (ng/ml)	2.5 ± 0.5	1.3 ± 0.2	3.1 ± 0.9	5.8. ± 0.9*	2.5 ± 0.5^†^	1.3 ± 0.8^†^	4.1 ± 0.9
Serum TC (mg/dl)	82 ± 5	85 ± 5	95 ± 4	117 ± 3	120 ± 6	109 ± 12	96 ± 7

Values are mean ± SEM. N = 4–11.

*TC* total cholesterol.

* P < 0.05 vs. LETO vehicle; ^†^P < 0.05 vs. OLETF vehicle.

In OGTTs, levels of blood glucose and serum insulin before and after glucose administration were higher in vehicle-treated OLETF than in LETO (Fig. 2b, c). Treatment with vildagliptin did not change glucose levels before and after glucose administration in OLETF (Fig. 2b). On the other hand, insulin level was significantly lower in the vildagliptin-treated OLETF than in the vehicle-treated OLETF (Fig. 2c).

### Cardiac function was not modified by vildagliptin or exenatide at baseline

Before induction of MI, heart rate was lower and left ventricular (LV) dimension was larger in OLETF than in LETO, though there were no differences in LV ejection fraction and fractional shortening between the two groups (Table 2). Treatment with vildagliptin, exenatide, or chloroquine did not alter these echocardiographic parameters in either LETO or OLETF. We tried to assess LV function at 12 h after coronary ligation also, but clear cardiac images for quantitative assessment could not be obtained at that time point because of intra-thoracic air, fluid and chest wall damage related to open-chest surgery.

**Table 2:** Echocardiographic data at baseline

	LETO				OLETF			
	Vehicle	Vildagliptin	Exenatide	Chloroquine	Vehicle	Vildagliptin	Exenatide	Vildagliptin + Chloroquine
HR (bpm)	384 ± 6	386 ± 7	385 ± 4	375 ± 6	312 ± 7*	316 ± 4*	315 ± 6*	324 ± 9*
LVEF (%)	69.4 ± 1.5	70.8 ± 1.3	68.4 ± 1.2	73.0 ± 1.3	65.4 ± 1.5	67.7 ± 0.7	68.5 ± 1.3	69.9 ± 1.3
FS (%)	35.0 ± 1.3	35.9 ± 1.0	34.0 ± 0.8	37.6 ± 1.5	32.1 ± 1.1	33.4 ± 0.5	34.4 ± 1.0	35.2 ± 1.1
IVST (mm)	1.72 ± 0.04	1.69 ± 0.02	1.76 ± 0.03	1.75 ± 0.04	1.81 ± 0.04	1.74 ± 0.03	1.73 ± 0.03	1.85 ± 0.02
PWT (mm)	1.66 ± 0.06	1.73 ± 0.05	1.64 ± 0.02	1.76 ± 0.06	1.85 ± 0.05	1.79 ± 0.04	1.83 ± 0.06	1.84 ± 0.03
LVEDD (mm)	7.04 ± 0.12	6.71 ± 0.19	7.11 ± 0.12	6.68 ± 0.12	8.05 ± 0.18*	7.69 ± 0.10*	7.88 ± 0.12*	7.65 ± 0.15*
LVESD (mm)	4.60 ± 0.15	4.34 ± 0.13	4.69 ± 0.09	4.16 ± 0.14	5.49 ± 0.17*	5.08 ± 0.10*	5.17 ± 0.12*	4.94 ± 0.07*
LVEDV (ml)	0.80 ± 0.04	0.75 ± 0.04	0.83 ± 0.04	0.69 ± 0.03	1.17 ± 0.07*	1.02 ± 0.04*	1.10 ± 0.04*	1.00 ± 0.06*
LVESV (ml)	0.26 ± 0.02	0.22 ± 0.01	0.26 ± 0.02	0.19 ± 0.02	0.41 ± 0.03*	0.37 ± 0.04*	0.35 ± 0.02*	0.30 ± 0.01*

Values are mean ± SEM. N = 12–33.

*HR* heart rate, *bpm* beats per minute, *LVEF* left ventricular ejection fraction, *FS* fractional shortening, *IVST* interventricular septal thickness, *PWT* posterior wall thickness, *LVEDD* left ventricular end-diastolic dimension, *LVESD* left ventricular end-systolic dimension, *LVEDV* left ventricular end-diastolic volume, *LVESV* left ventricular end-systolic volume.

* P < 0.05 vs. LETO Vehicle.

### Vildagliptin improved survival in OLETF in a chloroquine-sensitive manner

**Fig. 3:**
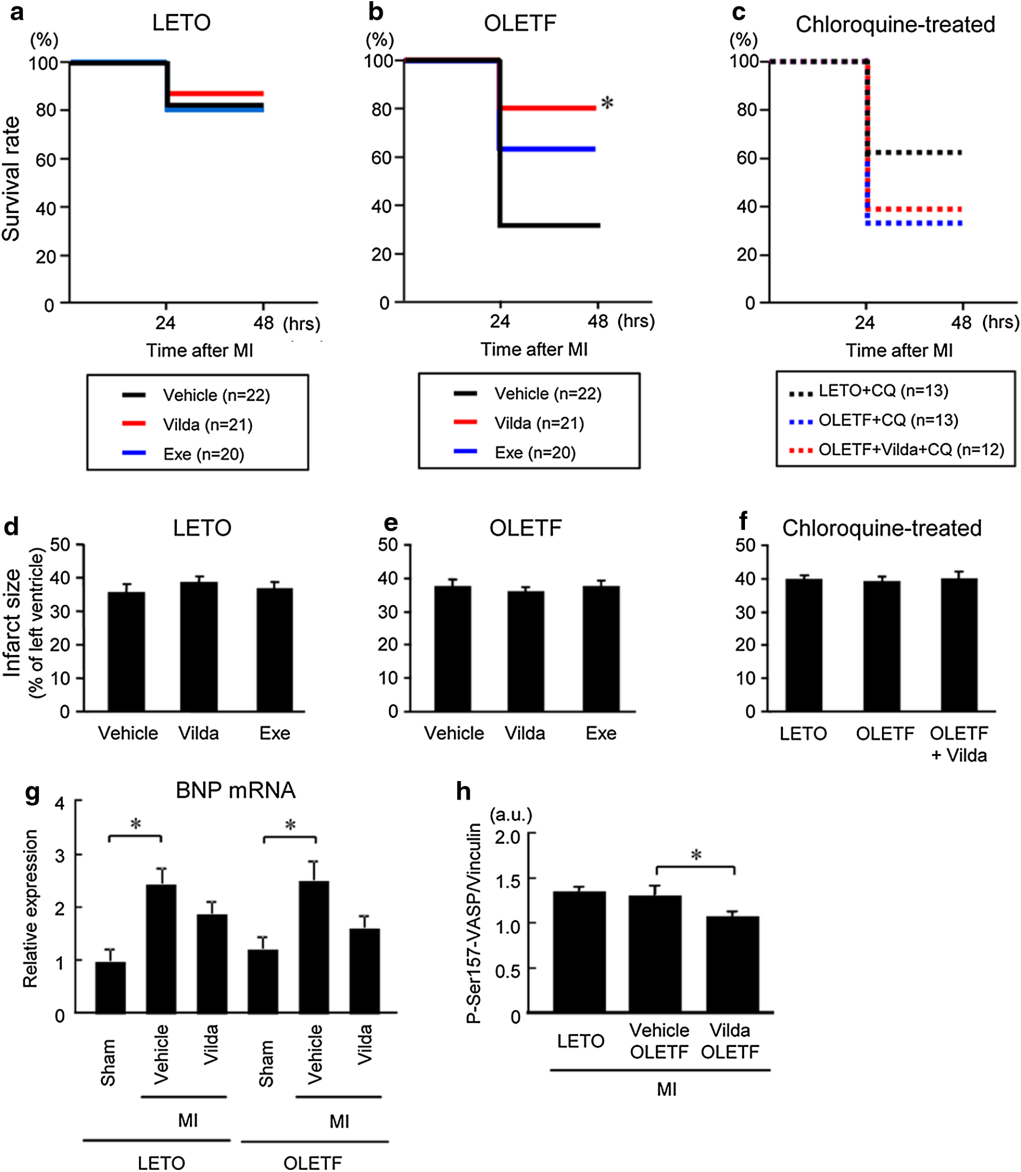
Effects of vildagliptin, exenatide, and chloroquine on survival after MI. Kaplan–Meier survival analysis of LETO (**a**), OLETF (**b**), and rats treated with chloroquine (**c**) after left coronary artery occlusion. *p < 0.05 vs. Vehicle-treated group. Infarct size measured at 48 h after MI in LETO (**d**), OLETF (**e**), and rats treated with chloroquine (**f**). **g** Quantification of BNP mRNA levels normalized to β-actin in the non-infarcted myocardium sampled 12 h after MI. N = 3–6 in each group. **h** Summary data of immunoblotting for phospho-Ser157 VASP in samples from LETO, vehicle- or vildagliptin-treated OLETF after MI. N = 9–12 in each group. *Vilda* vildagliptin, *Exe* exenatide, *CQ* chloroquine.

The survival rate during a period of 48 h after MI in LETO was 82%, which was comparable with the rate in our previous study [4]. Pretreatment with vildagliptin or exenatide for 2 weeks before MI did not affect the survival rate in LETO (86 and 80%, respectively) (Fig. 3a). As in our previous study [4], the survival rate was significantly lower in OLETF (32%, Fig. 3b) than that in LETO. Pretreatment of OLETF with vildagliptin significantly increased the survival rate (80%) to a level similar to that in LETO (Fig. 3b). Exenatide treatment tended to improve the survival rate in OLETF, but the difference did not reach statistical significance. In chloroquine-treated LETO, the survival rate (62%, Fig. 3c) tended to be lower than that in vehicle-treated LETO (Fig. 3a). Survival rates of chloroquine-treated OLETF were similar, regardless of vildagliptin treatment or no vildagliptin treatment (33 and 39%, respectively, Fig. 3c), to the survival rate in vehicle-treated OLETF (Fig. 3b). Autopsies of rats died within 48 h after MI revealed no case of cardiac rupture.

**Fig. 4:**
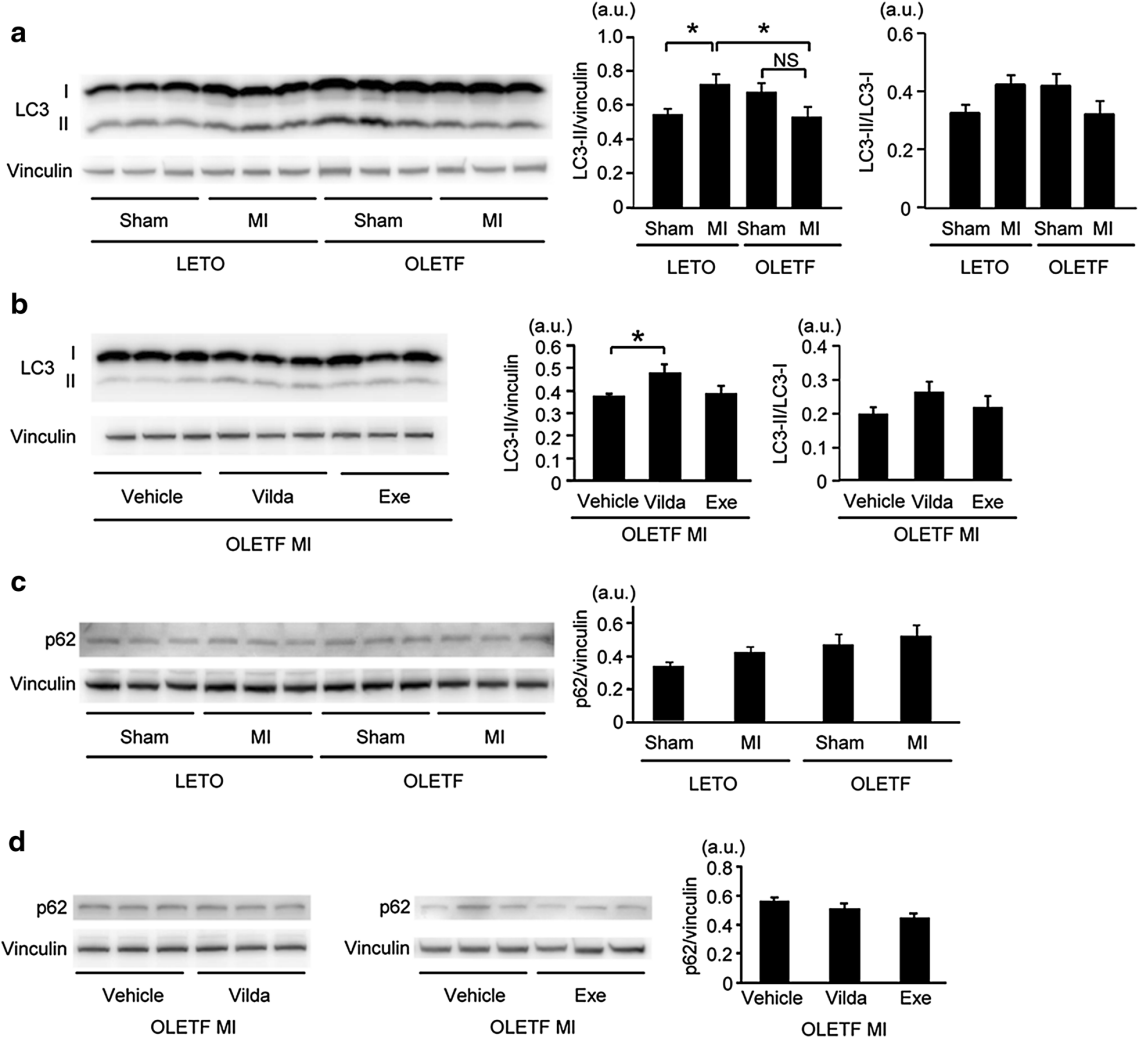
Vildagliptin restored autophagic induction in the non-infarcted area after MI in OLETF. **a** Representative images of Western blotting for LC3 protein (*left*) and summary data of LC3-II level and LC3-II/LC3-I ratio (*right*) in samples from sham-operated hearts (Sham) or the non-infarcted myocardium after MI in LETO and OLETF. **b** Representative images of Western blotting for LC3 protein (*left*) and summary data of LC3-II level and LC3-II/LC3-I ratio (*right*) in samples from the non-infarcted myocardium after MI in OLETF treated with the vehicle, vildagliptin (*Vilda*), or exenatide (*Exe*). **c** Representative blots (*left)* and summary data (*right*) of Western blotting for p62 protein in samples from sham-operated hearts (Sham) or the non-infarcted myocardium after MI in LETO and OLETF. **d **Representative blots (*left and middle*) and summary data (*right*) of Western blotting for p62 protein in OLETF treated with the vehicle, Vilda, or Exe after MI. N = 8–10 in each group. *p < 0.05. *a.u*. arbitrary units, *NS* not significant.

Infarct sizes 48 h after the permanent coronary occlusion were 35–40% of the left ventricle and were comparable among the treatment groups (Fig. 3d–f). In LETO, hemodynamic parameters (heart rate and blood pressures) at 12 h after MI in immunoblot experiments were comparable between the treatment groups (Table 3). Heart rates before and after MI and blood pressure at 12 h after MI were lower in OLETF than in LETO, but treatment with either vildagliptin or exenatide did not significantly change these hemodynamic parameters in OLETF. Taken together, these results indicate that difference in infarct size or hemodynamic response does not underlie the difference in survival rate among the treatment groups.

**Table 3:** Hemodynamic data 12 h after myocardial infarction

	LETO				OLETF			
	Sham	MI			Sham	MI		
	Vehicle	Vehicle	Vildagliptin	Exenatide	Vehicle	Vehicle	Vildagliptin	Exenatide
SBP (mmHg)	119 ± 5	105 ± 6	115 ± 4	114 ± 5	104 ± 6	90 ± 3*	96 ± 4*	93 ± 5*
DBP (mmHg)	88 ± 4	81 ± 6	85 ± 4	82 ± 6	73 ± 7	64 ± 3*	65 ± 4*	64 ± 4*
Heart rate (bpm)	423 ± 7	401 ± 8	410 ± 11	400 ± 10	332 ± 13*	321 ± 6*	331 ± 7*	326 ± 9*

Values are measn ± SE. N = 8–15.

*SBP* systolic blood pressure, *DBP* diastolic blood pressure, *bpm* beats per minute.

* P < 0.05 vs. LETO Sham.

To examine whether the loading condition in the non-infarcted region after MI was modulated by vildagliptin, we measured BNP mRNA level in the remote myocardium 12 h after MI (Fig. 3g). BNP mRNA level was significantly increased after MI in both LETO and OLEFT, but such an increase in BNP mRNA after MI was not observed in OLETF treated with vildagliptin (Fig. 3g). Since augmented adrenergic activity is one of the features of heart failure, we determined phosphorylation of vasodilator-stimulated phosphoprotein (VASP) at Ser157, a protein kianse A phosphorylation site, in the myocardium. The levels of phospho-VASP after MI were similar in LETO and OLETF, but treatment of OLETF with vildagliptin significantly decreased phospho-VASP levels (Fig. 3h). These findings suggest that vildagliptin attenuated both ventricular overloading and augmented adrenergic drive after MI in OLETF.

### Autophagic response in the non-infarcted region of the myocardium after MI was impaired in OLETF

Marker molecules of autophagic activities in the non-infarcted region of the heart after MI are shown in Fig. 4. LC3-II levels in the heart without infarction (i.e., sham operation) were similar in LETO and OLETF. LC3-II/vinculin ratio was significantly increased after MI in LETO, but such a response was not observed in OLETF (Fig. 4a). Changes in LC3-II/LC3-I ratio were similar to those in LC3-II/vinculin, though the difference did not reach statistical significance. Vildagliptin significantly increased LC3-II level and tended to increase LC3-II/LC3-I after MI in OLETF (Fig. 4b). Although p62 protein is often used as an index of autophagic flux [38], its level was not changed by MI in either LETO or OLETF (Fig. 4c). Neither vildagliptin nor exenatide changed p62 protein level after MI in OLETF (Fig. 4d). mRNA levels of p62 in the myocardium were also similar in LETO and OLETF regardless of MI and treatment with vildagliptin or exenatide (data not shown).

**Fig. 5:**
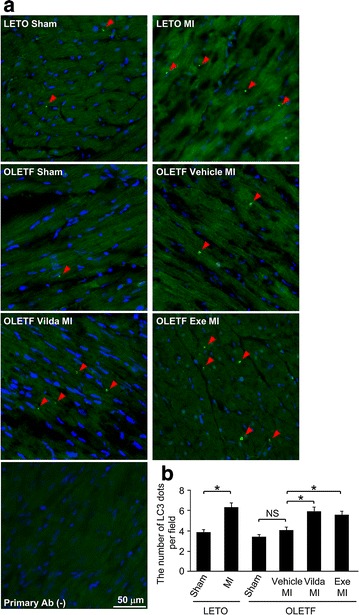
Immunofluorescent analysis of LC3 protein in the non-infarcted myocardium after MI. **a** Representative immunofluorescence images of LC3 protein in LV sections from the non-infarcted myocardial area after MI. The image without the primary antibody did not show any *green dots*. **b** Quantification of LC3 dots per field (568 µm × 426 µm). A total of 40 fields from five hearts were analyzed in each group. *p < 0.05. *Vilda* vildagliptin, *Exe* exenatide, *NS* not significant.

Autophagic activity in the non-infarcted region of the myocardium was assessed also by immunostaining of autophagosomes with anti-LC3 antibody (Fig. 5). In LETO, the number of LC3-positive dots was significantly increased after MI by 66%, but such an increase was not observed in OLETF. Not only vildagliptin but also exenatide significantly increased the number of LC3 dot after MI in OLETF (Fig. 5), indicating that vildagliptin and exenatide restored autophagic induction in the non-infarcted myocardium after MI in OLETF.

**Fig. 6:**
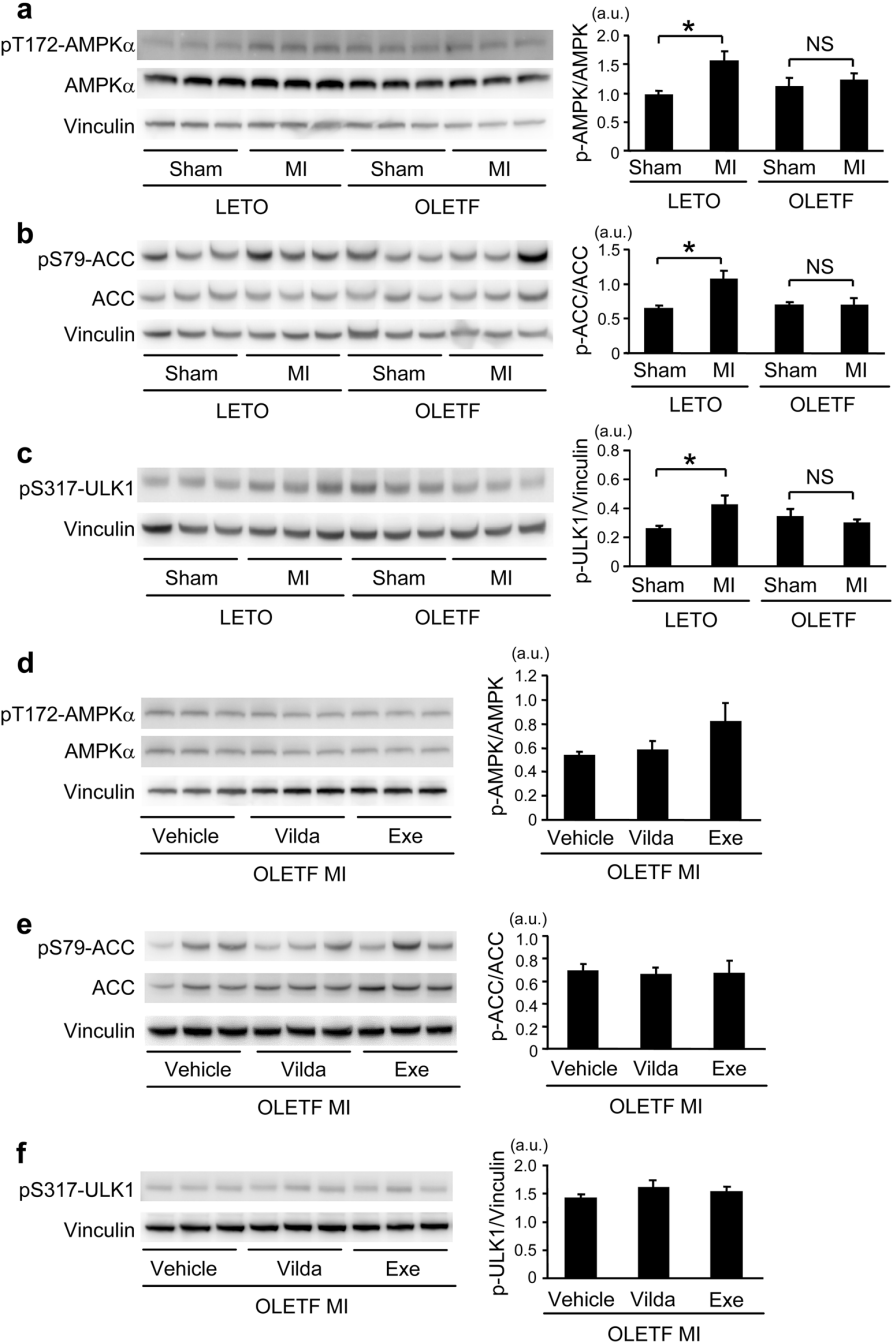
Analysis of the AMPK/ULK1 pathway. Representative images (*left*) and summary data (*right*) of Western blotting for phospho-Thr172 and total AMPKα (**a**), phospho-Ser79 and total acetyl-CoA carboxylase (ACC) (**b**), and phospho-Ser317-ULK1 (**c**) in samples from sham-operated heats or the non-infarcted myocardium after MI in LETO and OLETF. Representative blots (*left*) and summary data (*right*) of Western blotting for phospho-Thr172 and total AMPKα (**d**), phospho-Ser79 and total acetyl-CoA carboxylase (ACC) (**e**), and phospho-Ser317-ULK1 (**f**) in the non-infarcted myocardium after MI in OLETF treated with the vehicle, vildagliptin (*Vilda*), or exenatide (*Exe*). N = 9–10 in each group. *p < 0.05. *a.u.* arbitrary units, *NS* not significant.

### Activation of AMPK in response to MI was impaired in OLETF

Since AMPK is known to activate autophagy by phosphorylating ULK1 at Ser317 [25], we assessed AMPKα phosphorylation at Thr172 and phosphorylation of its downstream target proteins, acetyl-CoA carboxylase (ACC) and ULK1, in the non-infarcted region after MI (Fig. 6a–c). Levels of phospho-Thr172-AMPKα, phospho-Ser79-ACC and phospho-Ser317-ULK1 were significantly increased at 12 h after MI in LETO. In contrast, such responses of AMPKα, ACC and ULK1 were not detected in OLETF. Although vildagliptin and exenatide restored the increase in LC3-positive autophagosomes after MI in OLETF (Fig. 5), neither agent restored phosphorylation of Thr172-AMPKα, Ser79-ACC or Ser317-ULK1 after MI in OLETF (Fig. 6d–f).

**Fig. 7:**
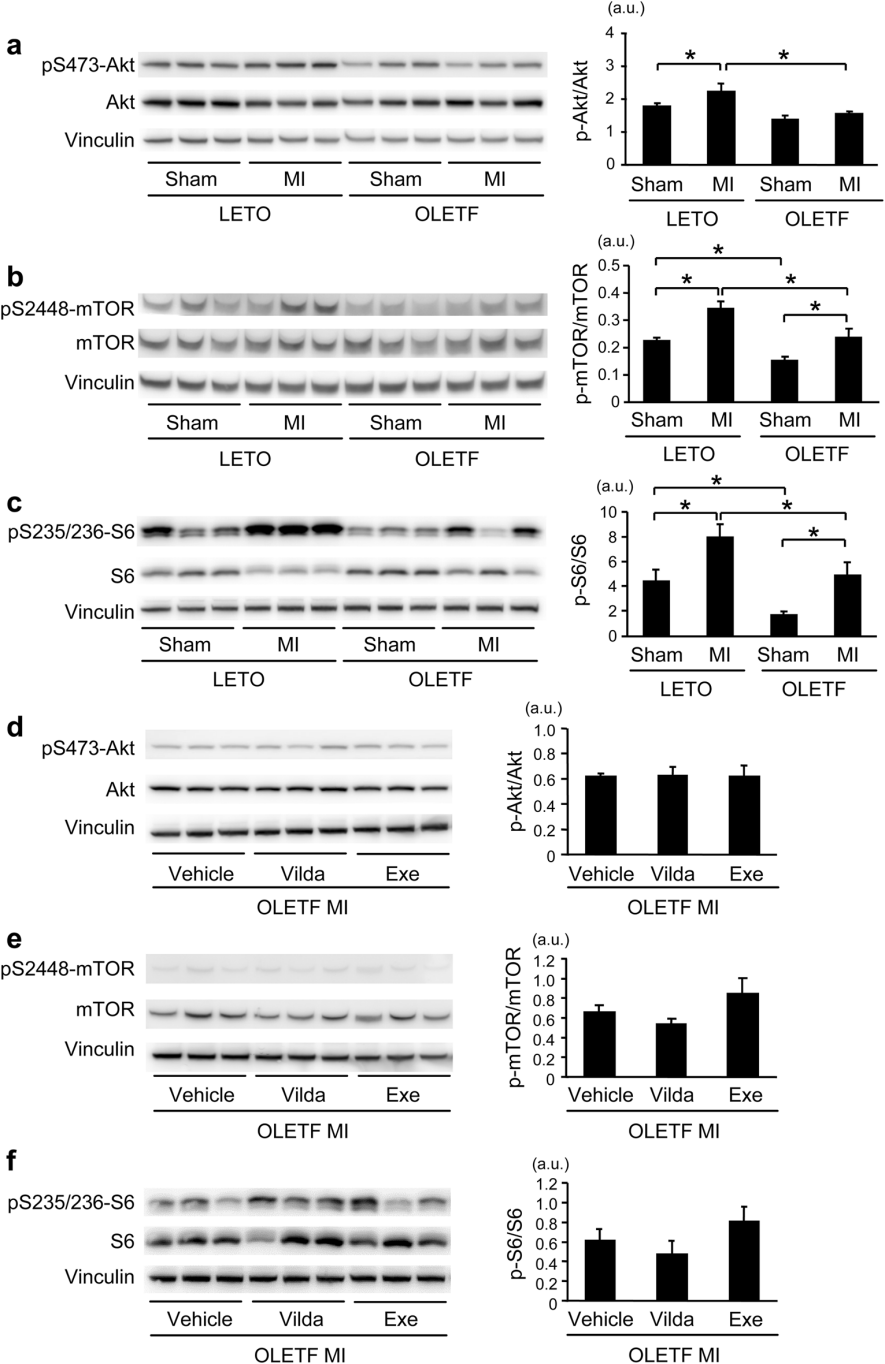
Analysis of Akt/mTORC1 activity. Representative blots (*left*) and summary data (*right*) of Western blotting for phospho-Ser473 and total Akt (**a**), phospho-Ser2448 and total mTOR (**b**), and phospho-Ser235/236 and total S6 (**c**) in samples from sham-operated heats or the non-infarcted myocardium after MI in LETO and OLETF. Representative blots (*left*) and summary data (*right*) of Western blotting for phospho-Ser473 and total Akt (**d**), phospho-Ser2448 and total mTOR (**e**), and phospho-Ser235/236 and total S6 (**f**) in the non-infarcted myocardium after MI in OLETF treated with the vehicle, vildagliptin (*Vilda*), or exenatide (*Exe*). N = 9–10 in each group. *p < 0.05. *a.u.* arbitrary units.

### Akt/mTORC1 activity after MI was attenuated in the OLETF hearts

**Fig. 8:**
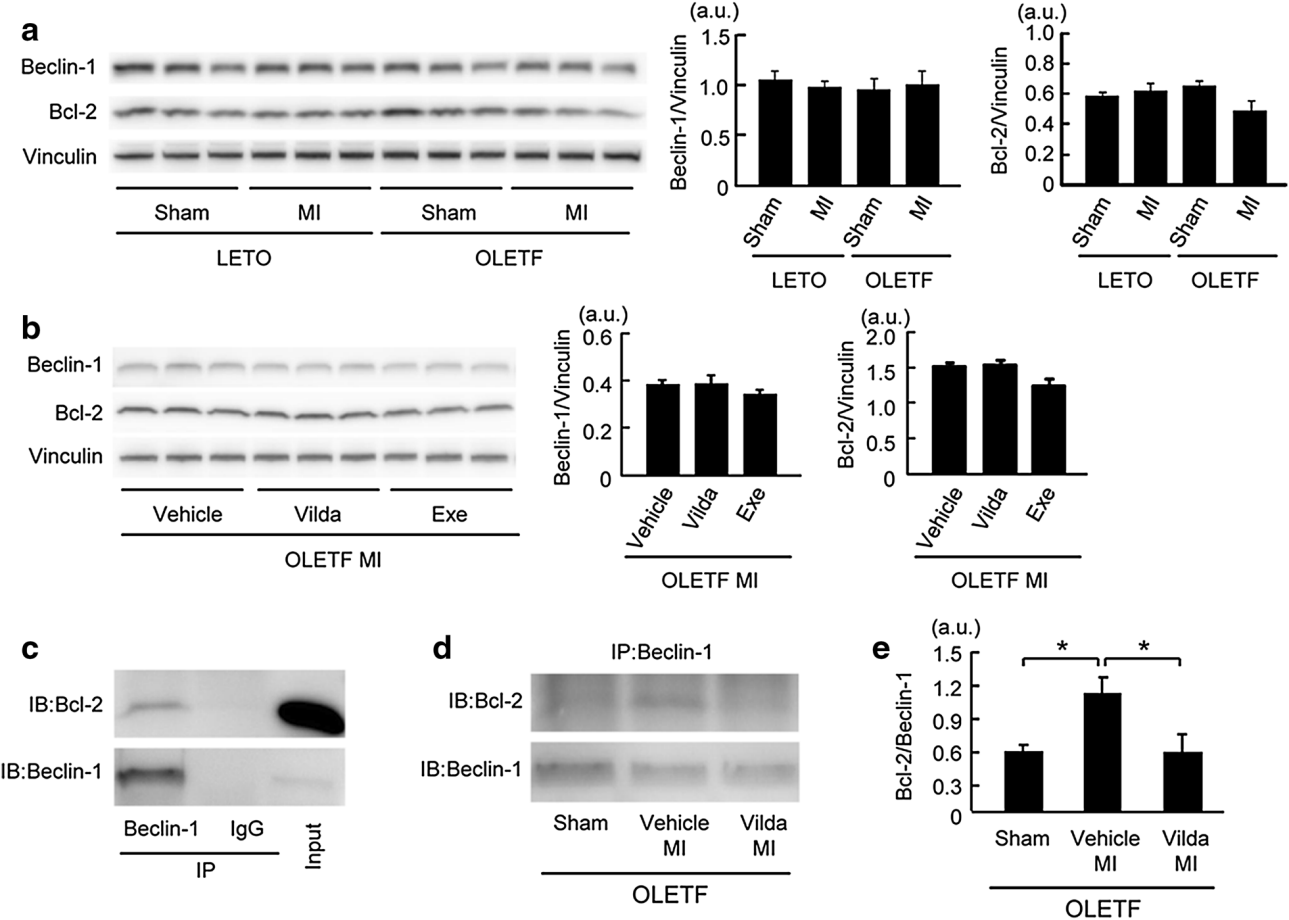
Effect of vildagliptin on Beclin-1/Bcl-2 interaction after MI in OLETF. **a** Representative images (*left*) and summary data (*right*) of Western blotting for Beclin-1 and Bcl-2 in samples from sham-operated heats or the non-infarcted myocardium after MI in LETO and OLETF. **b** Effects of vildagliptin (*Vilda*) and exenatide (*Exe*) on Beclin-1 and Bcl-2 levels in the non-infarcted myocardium after MI in OLETF. Representative immunoblotting images (*left*) and summary data (*right*) are shown. **c** Myocardial lysates were immunoprecipitated (IP) with anti-Beclin-1 antibody or rabbit IgG followed by immunoblotting with anti-Bcl-2 and Beclin-1 antibodies. **d**, **e** Beclin-1/Bcl-2 interaction was increased after MI in OLETF, which was attenuated by vildagliptin (*Vilda*). N = 5 in each group. *p < 0.05. *a.u.* arbitrary units.

Alterations in Akt/mTORC1 signaling, a negative regulatory mechanism of autophagy [25], in OLETF were examined by immunoblotting. The level of Ser473-Akt phosphorylation was significantly elevated after MI in the non-infarcted myocardium in LETO (Fig. 7a). The phosphorylation was associated with increases in levels of phospho-mTOR at Ser2448 and phospho-S6 at Ser235/236 (Fig. 7b, c). However, such an activation of Akt after MI was not observed in OLETF; phospho-Akt levels were similar in sham-operated and MI-induced OLETF (Fig. 7a). Phospho-mTOR and phospho-S6 levels were increased after MI in OLETF, but their levels remained significantly lower than those in LETO (Fig. 8b, c). Neither vildagliptin nor exenatide increased phosphorylation of Ser473-Akt, Ser2448-mTOR, and Ser235/236-S6 after MI in OLETF (Fig. 7d–f).

### Beclin-1–Bcl-2 interaction was enhanced in OLETF

Since vildagliptin improved the response of autophagic activity to MI in OLETF (Figs. 4, 5) without normalization of AMPK phosphorylation and Akt/mTORC1 signaling (Figs. 6, 7), we examined whether Beclin-1–Bcl-2 interaction is modified in OLETF or by vildagliptin treatment. Beclin-1 is an essential component for activation of autophagy, and its autophagy-promoting activity is inhibited by binding to an anti-apoptotic protein, Bcl-2 [39]. There were no significant differences in Beclin-1 and Bcl-2 protein levels between LETO and OLETF regardless of MI (Fig. 8a), and neither vildagliptin nor exenatide changed levels of these proteins in MI-induced OLETF (Fig. 8b). However, Beclin-1–Bcl-2 interaction was significantly augmented in OLETF with MI compared to that in OLETF with a sham operation, and their interaction was attenuated by vildagliptin (Fig. 8c–e).

## Discussion

In the present study, treatment with vildagliptin at a dose that did not lower plasma glucose level significantly improved survival of OLETF after acute MI (Figs. 2, 3). By using telemetric monitoring of heart rate and blood pressure, we previously demonstrated that increased mortality during the acute phase after MI in OLETF is due to progressive heart failure but not lethal arrhythmia [4]. Although vildagliptin did not modify ventricular function under baseline conditions (Table 2), it suppressed MI-induced upregulation of BNP expression and cardiac adrenergic activity in OLETF (Fig. 3g, h). Thus, suppression of heart failure progression after MI is the most likely explanation for reduction in acute mortality after MI in OLETF by vildagliptin.

Serum active GLP-1 level was elevated by vildagliptin (Fig. 2a), suggesting that GLP-1 mediated the improved survival by vildagliptin. On the other hand, treatment with a GLP-1 analog, exenatide, tended to improve post-MI survival in OLETF, but the effect was statistically insignificant. We do not have a clear explanation for the different outcomes in the vildaglitpin-treated and exenatide-treated groups. However, a difference in the mechanism of cardioprotection is a possibility. The GLP-1 receptor is localized in the cardiomyocyte [40], but a  recent study has shown that a GLP-1 receptor agonist provides cardioprotection by a mechanism independent of the GLP-1 receptor in the cardiomyocyte [31]. On the other hand, DPP-4 is involved in degradation of multiple peptides such as substance P and stromal cell-derived factor-1 [27, 41], and these properties of DPP-4 inhibitors might underlie the differences in survival rate (Fig. 3b) and changes in LC3-II protein level (Fig. 4b) after MI between vildagliptin-treated and exenatide-treated OLETF.

In the literature, a study by French et al. [42] is the only study in which the changes in autophagy after MI in diabetic mice and non-diabetic mice were compared. In that study, autophagic activity was not increased after MI not only in the diabetic heart but also in the non-diabetic heart. The negative results are in contrast to results of several studies showing that autophagic activity was increased after MI in the healthy control myocardium [5, 8–12]. The reason why French et al. could not detect an alteration of autophagy after MI even in the non-diabetic myocardium is unclear, but use of entire risk zone tissue consisting of infarcted and non-infarcted cells might have obscured changes in autophagy in the viable myocardium after MI.

We focused on the non-ischemic region of the infarcted heart since that region plays a crucial role in compensation for the lost function of the infarcted region and undergoes adaptive and maladaptive post-MI remodeling [43, 44]. There was no significant difference in LC3-II or p62 levels or LC3-positive autophagosomes between LETO and OLETF under baseline conditions (i.e., sham-operated groups in Figs. 4, 5), suggesting that autophagic activities were similar in the diabetic myocardium and non-diabetic myocardium under non-stressed conditions. However, there was a significant difference between LETO and OLETF in autophagic response after MI. In LETO, an increase in autophagic activity was observed in the non-infarct region at 12 h after MI (Figs. 4, 5). A protective role of the increase in autophagic activity after MI has been demonstrated by findings that inhibition of autophagy by bafilomycin A or genetic deletion of beclin-1 aggravated remodeling and dysfunction of the ventricle after MI [8, 11]. Importantly, the mortality rate after MI in OLETF was not further increased by inhibiting autophagy with chloroquine (Fig. 3). These results support the notion that impaired autophagic response in the non-infarcted region of the infarcted heart contributes to increase in acute mortality after MI in OLETF.

There are two possible mechanisms for suppression of heart failure by autophagy: reduction of reactive oxygen species (ROS) production and improvement of myocardial energy status. Damaged organelles participating in ROS generation, including mitochondria, are sequestrated and removed by the autophagic process, and autophagy plays a role in suppression of ROS generation [17, 45]. In fact, ROS generation from damaged mitochondria is involved in exacerbation of ventricular dysfunction [46]. An impact of autophagy on myocardial energy status has been shown by findings that myocardial ATP content after MI was increased by augmentation of autophagy [9]. Diabetes impairs mechanisms regulating ATP supply, and our recent study [33] showed that reduced myocardial reserve of ATP supply, leading to diastolic dysfunction, was disclosed by increased afterload in OLETF. How impaired response of autophagy relates to dysregulation of ATP supply mechanisms in diabetic hearts may warrant further investigation.

The results of the present study supported the notion that activation of autophagy by vildagliptin during the acute phase after MI contributed to improved survival in OLETF. However, roles of autophagy in the heart may be different depending on the phase and type of cardiac stress. Matsui et al. [5] showed that autophagy is protective for cardiomyocyte survival during ischemia but is rather detrimental during reperfusion. Zhu et al. [47] showed that sustained activation of autophagy during pressure overload is detrimental to cell survival. They showed that cardiac function after thoracic aortic banding was preserved in beclin-1 heterozygous knockout mice, whereas cardiomyocyte-specific overexpression of beclin-1 worsened cardiac function. In contrast, sustained elevation of autophagy may be protective for post-infarcted ventricular function and remodeling. Suppression of autophagy by bafilomycin A1 or chloroquine has been shown to exacerbate cardiac function after MI, but activation of autophagy by an mTORC1 inhibitor, rapamycin, or by caloric restriction was protective [8, 12]. Maejima et al. [11] also reported that cardiac function at 6 weeks after MI was worsened in beclin-1 heterozygous knockout mice. Since the survival rate of OLETF at 48 h after MI was only 32% (Fig. 3b), we did not include assessment of autophagic activity at the later phase after MI in OLETF in the present study.

Streptozotocin-induced diabetes and high-fat diet have been shown to reduce LC3-II in the myocardium, which was associated with suppressed phosphorylation of AMPK, a positive regulator of autophagy [16, 18]. In contrast, LC3-II, p62 or AMPK phosphorylation in the heart without MI was not different between OLETF and LETO in this study. However, Lee et al. [48] reported 50% reduction in AMPK phosphorylation in the myocardium of OLETF at the age of 28 weeks. A possible explanation for the discrepant results is more advanced stage of T2DM in OLETF in the study by Lee et al. [48]. Despite similar ages, OLETF in their study had slightly larger body weight compared with that in this study and showed significantly increased interstitial fibrosis in the heart [48], though such an increased collagen deposition in the myocardium was not detected by histochemistry or determination of mRNA levels of collagens I and III in OLETF used in our studies [4, 33]. Some difference in rearing conditions (possibly the amount or calories per volume of the chow provided) might underlie the difference in the phenotype of OLETF at similar ages. Nevertheless, it is possible that suppression of baseline AMPK phosphorylation and autophagy occurs in OLETF at an advanced stage of T2DM.

Increased AMPK phosphorylation with or without suppressed mTOR phosphorylation was associated with promotion of autophagy after MI in non-diabetic mice [9, 16]. The responses of autophagy, AMPK and mTOR to MI were confirmed in LETO (Figs. 4, 5, 6, 7). However, in OLETF, phosphorylation of AMPK was not increased and activation of the mTOR/S6 pathway was 60–70% of that in LETO (Figs. 6, 7). In addition, we found that interaction of Beclin-1 and Bcl-2, which reportedly inhibits Beclin-1-dependent autophagy [11, 15, 39], was significantly increased in the myocardium of OLETF (Fig. 8). Restoration of the adaptive responses of both LC3-II and autophagosomes after MI in OLETF by vildagliptin was associated with suppression of Beclin-1–Bcl-2 interaction but not with improved phosphorylation of AMPK, mTOR or S6. These findings suggest that increased Beclin-1–Bcl-2 interaction was responsible for T2DM-induced loss of adaptive autophagy in the non-ischemic myocardium after MI.

How vildagliptin suppressed Becin-1–Bcl-2 interaction in the myocardium of OLETF remains unclear. Among molecules that regulate Beclin-1–Bcl-2 interaction, AMPK-JNK activation has been reported to induce disruption of Beclin-1–Bcl-2 interaction through phosphorylation of Bcl-2 at Ser70 [15], whereas activation of mammalian sterile 20-like kinase 1 (Mst1) promoted Beclin-1/Bcl-2 interaction by phosphorylation of Beclin-1 at Thr108 [11]. In this study, vildagliptin did not restore phosphorylation of AMPK (Fig. 6) or JNK (data not shown). Hence, there is the possibility that vildagliptin suppressed Mst1 expression or activity, preventing interaction of Beclin-1 and Bcl-2 in the myocardium of OLETF. Unfortunately, we could not examine this possibility since phospho-Mst1 (Thr183) protein in the myocardium of OLETF could not be detected by use of commercially available antibodies.

Contrary to our expectations, the dose of vildagliptin we used in the present study was not sufficient for reducing glucose levels in OLETF (Table 1; Fig. 2b), though the dose of vildagliptin increased serum active GLP-1 level in OLETF (Fig. 2a). Therefore, the effects of vildagliptin on cardiac autophagy and mortality cannot be explained by its effect on glycemic control. Although DPP-4 inhibitors are known to enhance glucose-stimulated insulin release [49], reduction in plasma insulin level by DPP-4 inhibitors together with improved metabolic parameters has also been reported [50, 51]. It is notable that DPP-4 inhibitors (saxagliptin and alogliptin) failed to reduce cardiovascular mortality in diabetic patients at high cardiovascular risk in two large clinical trials [52, 53]. The effect of vildagliptin on acute mortality after MI in this study (Fig. 3) is difficult to easily reconcile with the negative results in the clinical trials [52, 53]. However, there is the possibility that the optimal dose of DPP-4 for cardiprotection is lower than that for glycemic control.

## Conclusions

Treatment with vildagliptin at a dose that elevated serum GLP-1 without normalization of plasma glucose level reduced acute mortality after MI in a rat model of T2DM to the level in non-diabetic controls. The beneficial effect of vildagliptin was sensitive to chloroquine and closely associated with restoration of the autophagic response in the non-infarcted myocardium to MI, suggesting an involvement of impaired autophagy in T2DM-induced increase in post-MI mortality.

## Additional file

Additional file 1:
**Table S1.** **Antibodies.** Description of data: a list of antibodies used in this study.
